# Supplementary material for: SOX2 Is a Univocal Marker for Human Oral Mucosa Epithelium Useful in Post-COMET Patient Characterization
Source: Int J Mol Sci. 2022 May 21;23(10):5785. doi: 10.3390/ijms23105785 (PMC9144017; doi:10.3390/ijms23105785)

Supplementary Materials and Data

Supplementary Data

Table S1: Strains and number of holoclones analyzed. The holoclones analyzed by microarray derived from two strains for each epithelium. Abbreviations: CON, conjunctiva; LE, limbus; MO, oral mucosa.

Epithelium	Strain	N. of holoclones
**Conjunctiva**	CON-89	6
	CON-90	3
**Limbus**	LE-51	2
	LE-113	6
**Oral Mucosa**	MO-14	6
	MO-34	9

Supplementary Materials

Cell cultures

Samples derived from the three epithelia were treated with trypsin (0.05% trypsin and 0.01% EDTA) at 37°C for about 120 min. Cells were collected every 30 mins and then seeded on lethally irradiated 3T3-J2 feeder layer, then cultured in 5% CO_2. Culture medium used was DMEM and Ham's F12 media (2:1 mixture) containing FBS (10%) and penicillin/streptomycin, insulin (5 µg/mL), adenine (0.18 mM), hydrocortisone (0.4 µg/mL), cholera toxin (0.1 nM), triiodothyronine (2 nM), glutamine (4 mM). Epidermal growth factor was added at 10 ng/mL beginning at the first feeding, 3 days after plating. Cultures were then fed every other day. Subconfluent primary cultures were passaged at a density of 6-8.3x10^3 cells/cm^2 and cultured as above.

For the stratification experiment, oral mucosa keratinocytes were cultured until confluence and then kept stratifying for 7 days, changing the media every other day. Growth was checked under the light microscope, and images were taken.

Clonal analysis and Colony Forming Efficiency assay

Sub-confluent epithelial cultures were trypsinized, serially diluted and plated in 96-well plates (0.5 cells per well). After 7 days of cultivation, single clones were identified under an inverted microscope and trypsinized. One-quarter of the clone was used for Colony Forming Efficiency (CFE) assay. It was cultured for 12 days onto a 100-mm (indicator) dish, which was then fixed and stained with rhodamine B to classify clonal type [31]. The remaining three-quarters of the clone was subcultured for proteins and RNA extraction and further analysis.

Real-time RT-PCR

Total RNA was isolated with the Invitrogen™ PureLink™ RNA Micro Scale Kit (Thermo Fisher), according to the manufacturer's protocol. cDNA was synthesized using SuperScript IV VILO Master Mix (Thermo Fisher). Real Time quantitative RT-PCR was performed by using TaqMan assays for PAX6 (Hs00240871_m1), SOX2 (Hs01053049_s1) and GAPDH (Hs99999905_m1), Taqman Fast Advanced Master Mix (all by Thermo Fisher) and the 7900HT Fast Real-Time PCR System (Applied Biosystems). The expression of target genes was normalized to the level of

GAPDH in the same cDNA by using the $2^{-\Delta\Delta CT}$ quantification. For statistical analysis Mann-Whitney test was applied.

Immunohistochemistry

Table S2: Primary Antibodies used for IF or IHC.

Target	Clone or n. of catalogue	Company	Dilution
**SOX2 (IF)**	D6D9	Cell Signalling	1:100
**SOX2 (IHC)**	SP-76	Cell Marque	ready-to-use
**PAX6 (IF)(IHC)**	Poly19013	Biolegend	1:3000 (IHC 1:500)
**Keratin 3-76 (IF)**	AE5	Progen	1:100
**Keratin 3-2p (IHC)**	AE5	Santa Cruz	1:300
**Keratin 13 (IF)**	GP-K13	Progen	1:100
**Keratin 13 (IHC)**	EPR3672	Abcam	1:100
**Keratin 12 (IHC)**	EPR17882	Abcam	1:1000

Western blot analyses

For protein extraction, pelleted cells were lysate with RIPA buffer at 4°C. Equal amounts of proteins were electrophoresed on NuPAGE (Invitrogen) 4-12% bis-tris sodium dodecyl sulfate-polyacrylamide gels under reducing conditions and transferred to nitrocellulose membranes and then blocked with 5% milk in PBS-Tween 0.1% buffer. Immunoreactions were performed using different antibodies diluted in blocking solution as listed in Table S3. Protein detection was carried out using a chemiluminescent labelling reagent (SuperSignal West Pico Chemiluminescent Substrate; Thermo Scientific).

Table S3: Primary Antibodies used for WB.

Target	Clone or n. of catalogue	Company	WB dilution
**GAPDH**	6C5	Abcam	1:10000
**Involucrin**	SY5	Leica Microsystems	1:10000
**Keratin 13**	EPR3672	Abcam	1:1000
**PAX6**	Poly19013	Biolegend	1:2000
**p63-α**	custom	PRIMM	1:5000
**SOX2**	D6D9	Cell Signalling	1:200

Supplementary Figures

Original Images for Blots

Blot Figure 3D

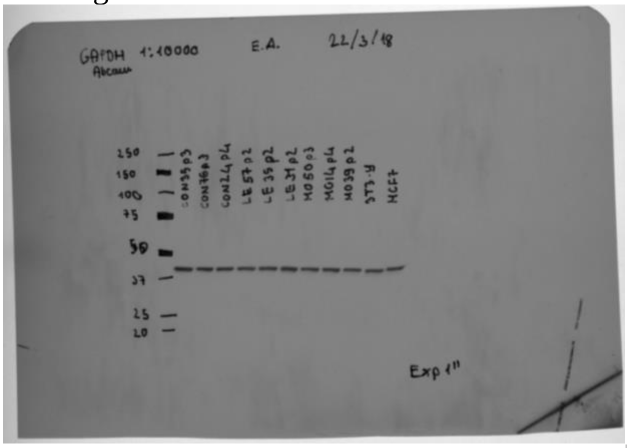

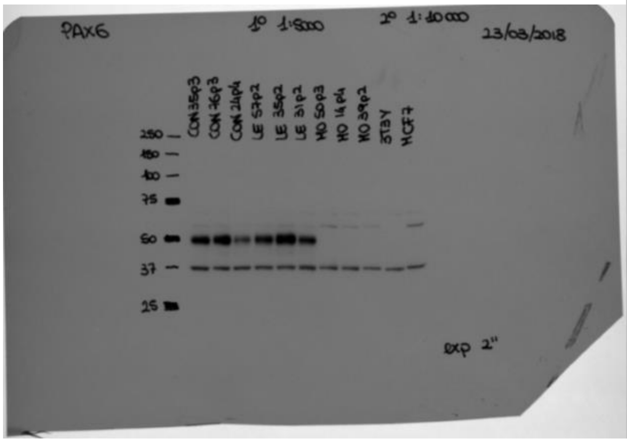

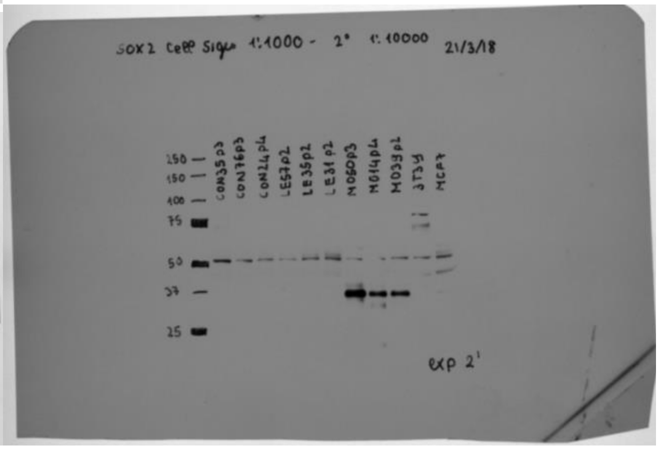

Blot Figure 3E

Order of samples (from left to right):

- MO14 p4 subconfluence
- MO14 p4 stratified
- MO50 p2 subconfluence
- MO50 p2 stratified
- MO39 p4 subconfluence
- MO39 p4 stratified
- LE31 p2 subconfluence
- LE31 p2 stratified
- 3T3
- UMSCC-14c

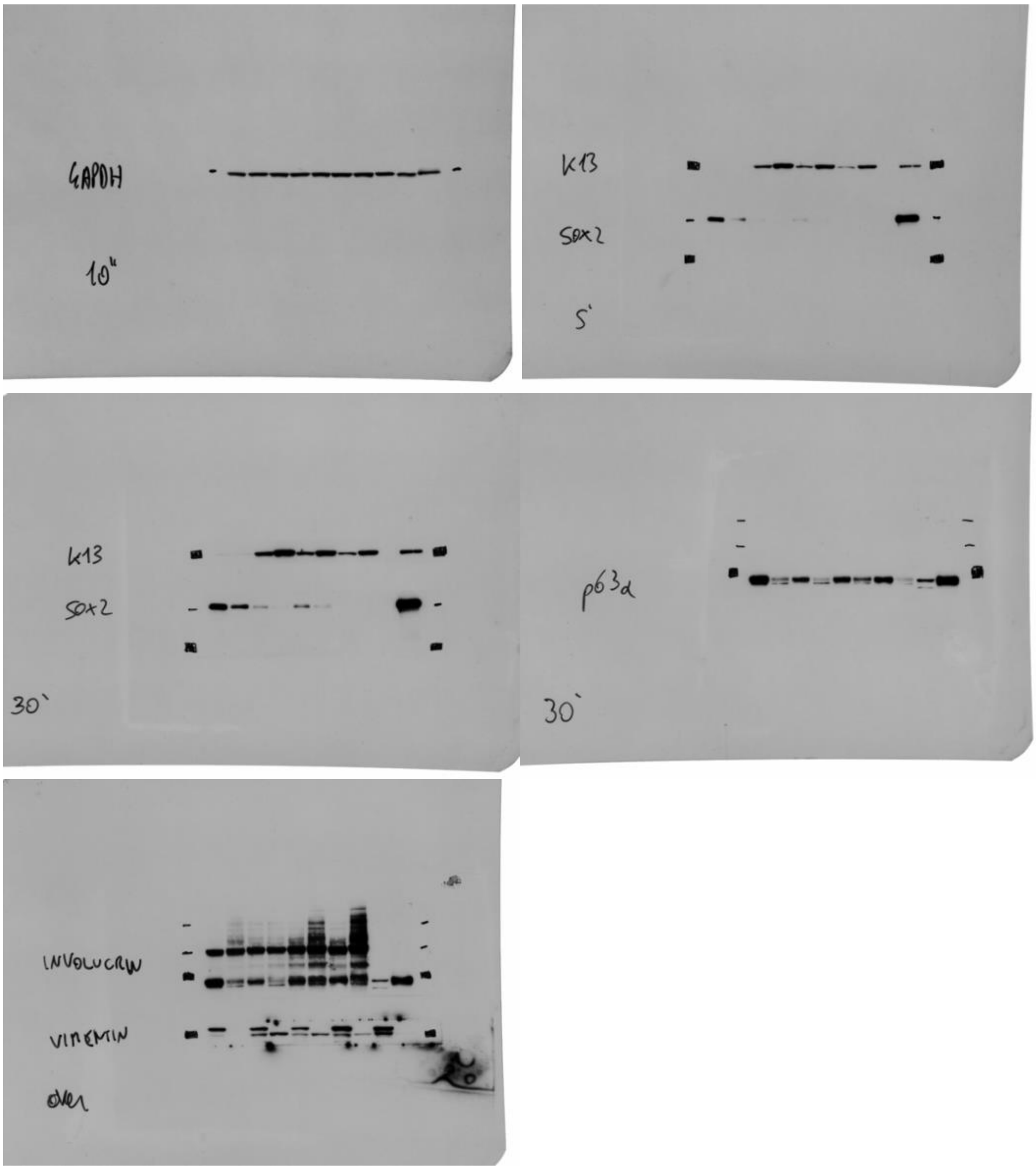

Supplement: Supplementary file 1 [file ijms-23-05785-s001.zip › Supplementary Materials and Data REV. 17-5-22.pdf]
