# Supplementary figures and images for: SOX2 Is a Univocal Marker for Human Oral Mucosa Epithelium Useful in Post-COMET Patient Characterization
Source: Int J Mol Sci. 2022 May 21;23(10):5785. doi: 10.3390/ijms23105785 (PMC9144017; doi:10.3390/ijms23105785)

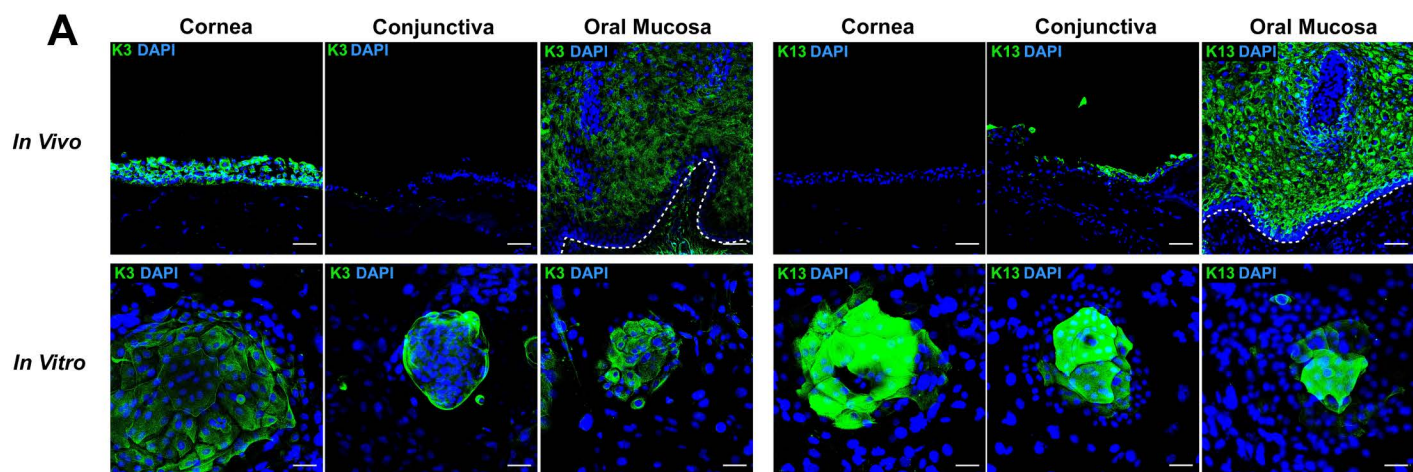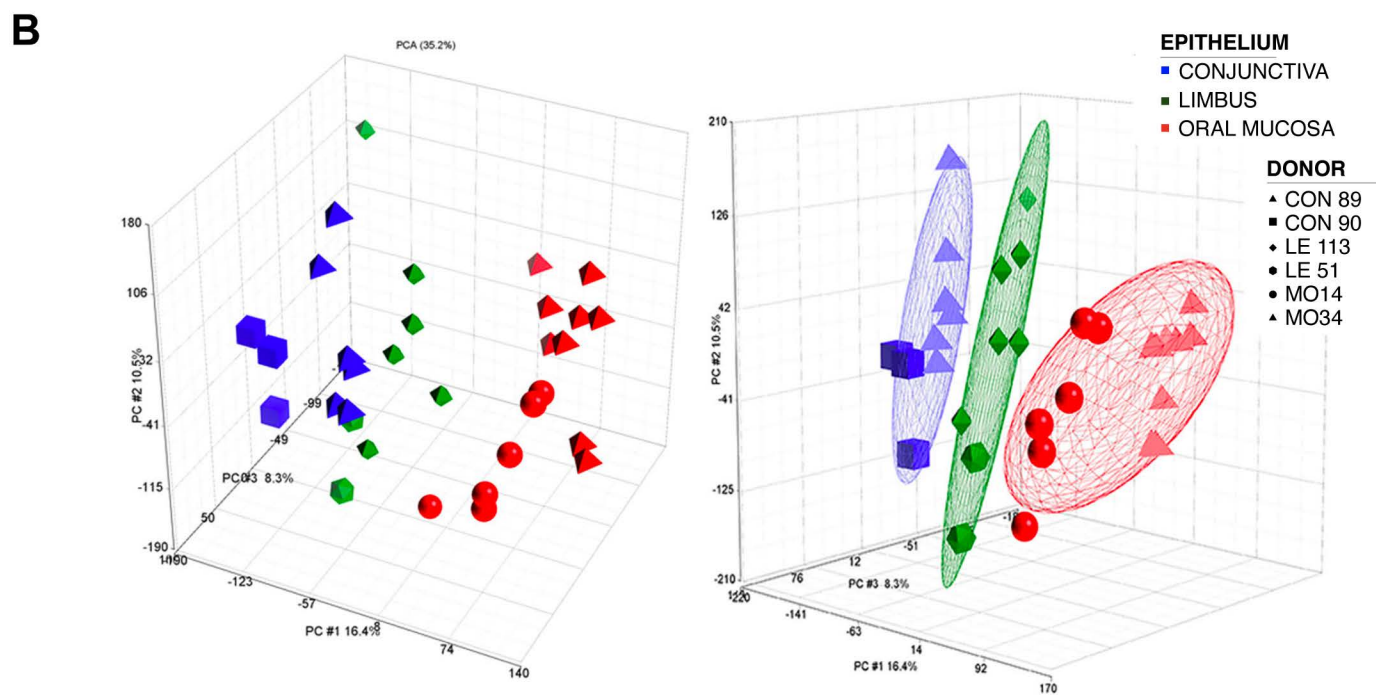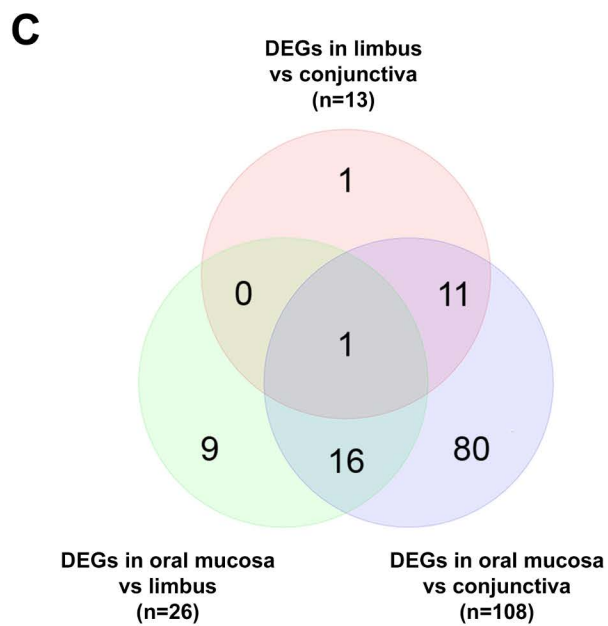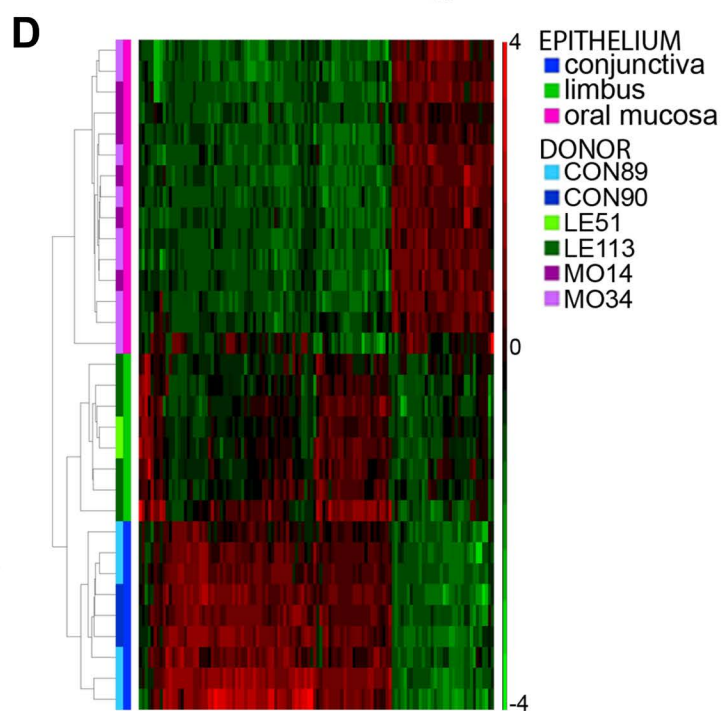

Supplement: Supplementary file 1 [file ijms-23-05785-s001.zip › Figure 1_V2-01.pdf]

**A**

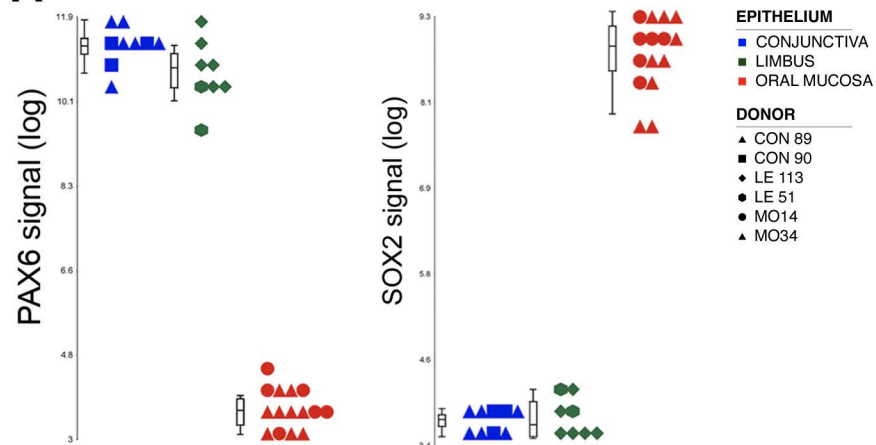

**B**

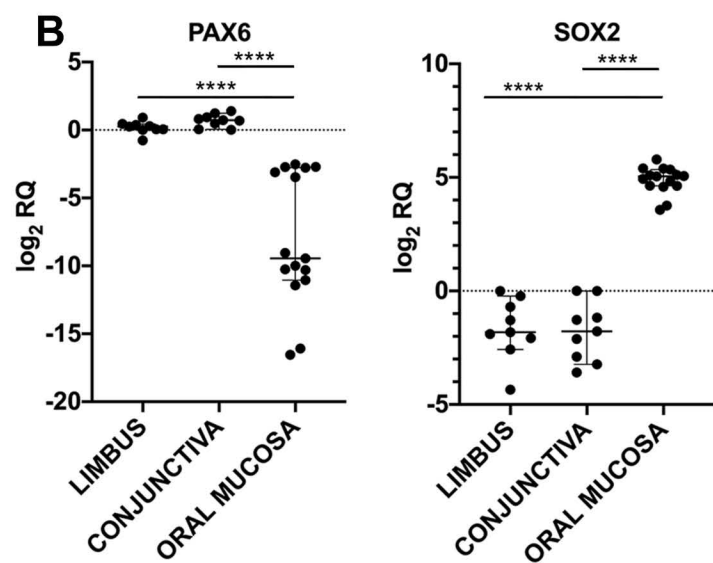

Supplement: Supplementary file 1 [file ijms-23-05785-s001.zip › Figure 2_V2-01.pdf]

**A**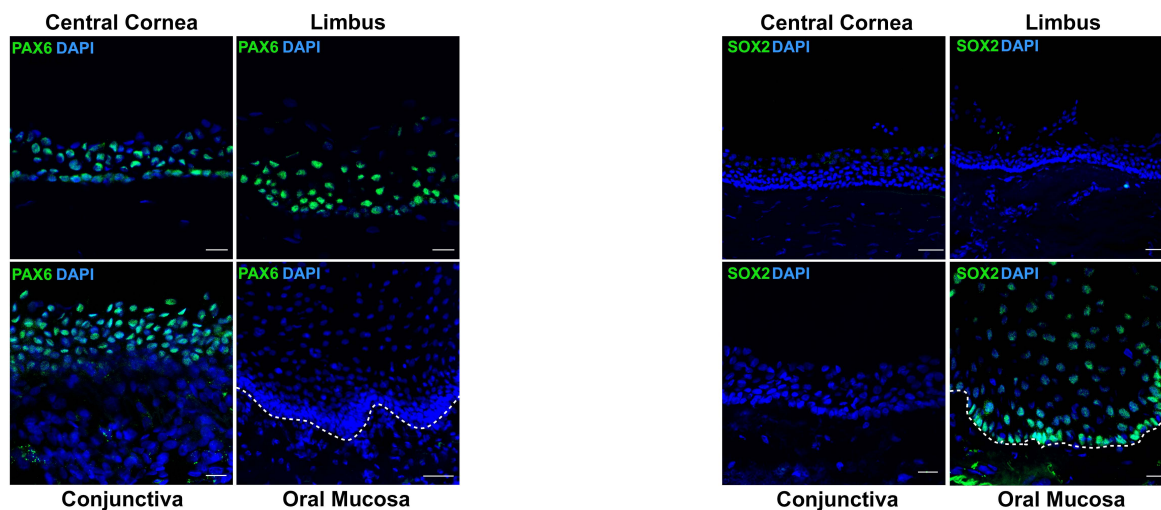**B**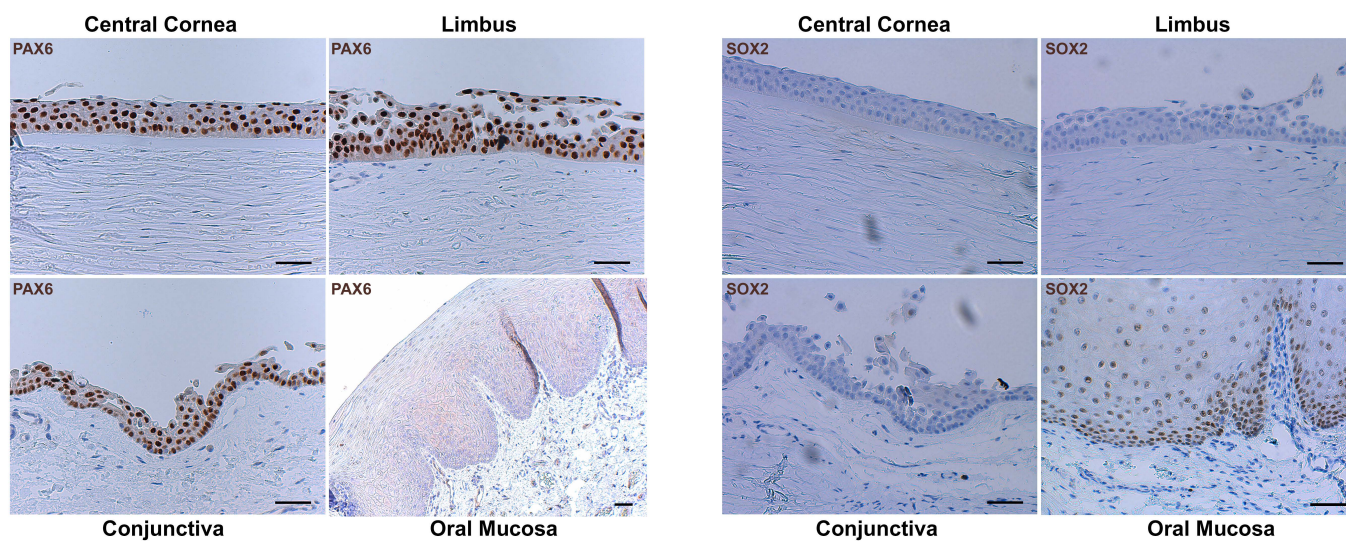**C**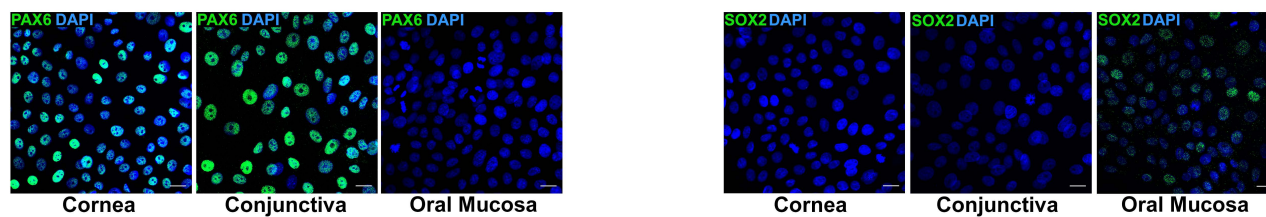**D**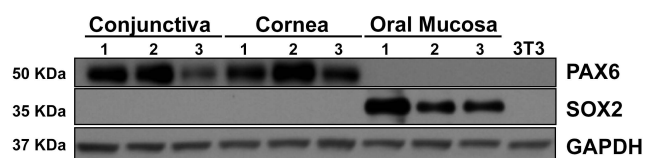**E**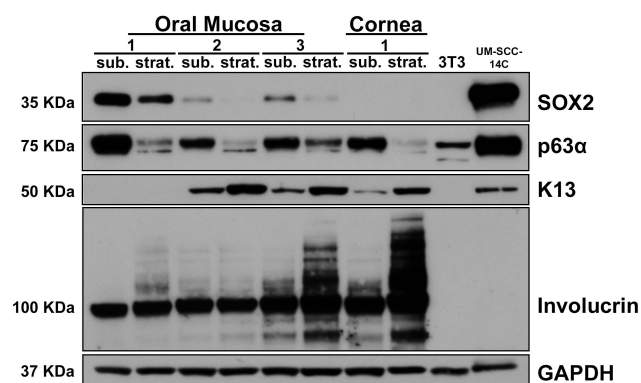

Supplement: Supplementary file 1 [file ijms-23-05785-s001.zip › Figure 3.pdf]

**A**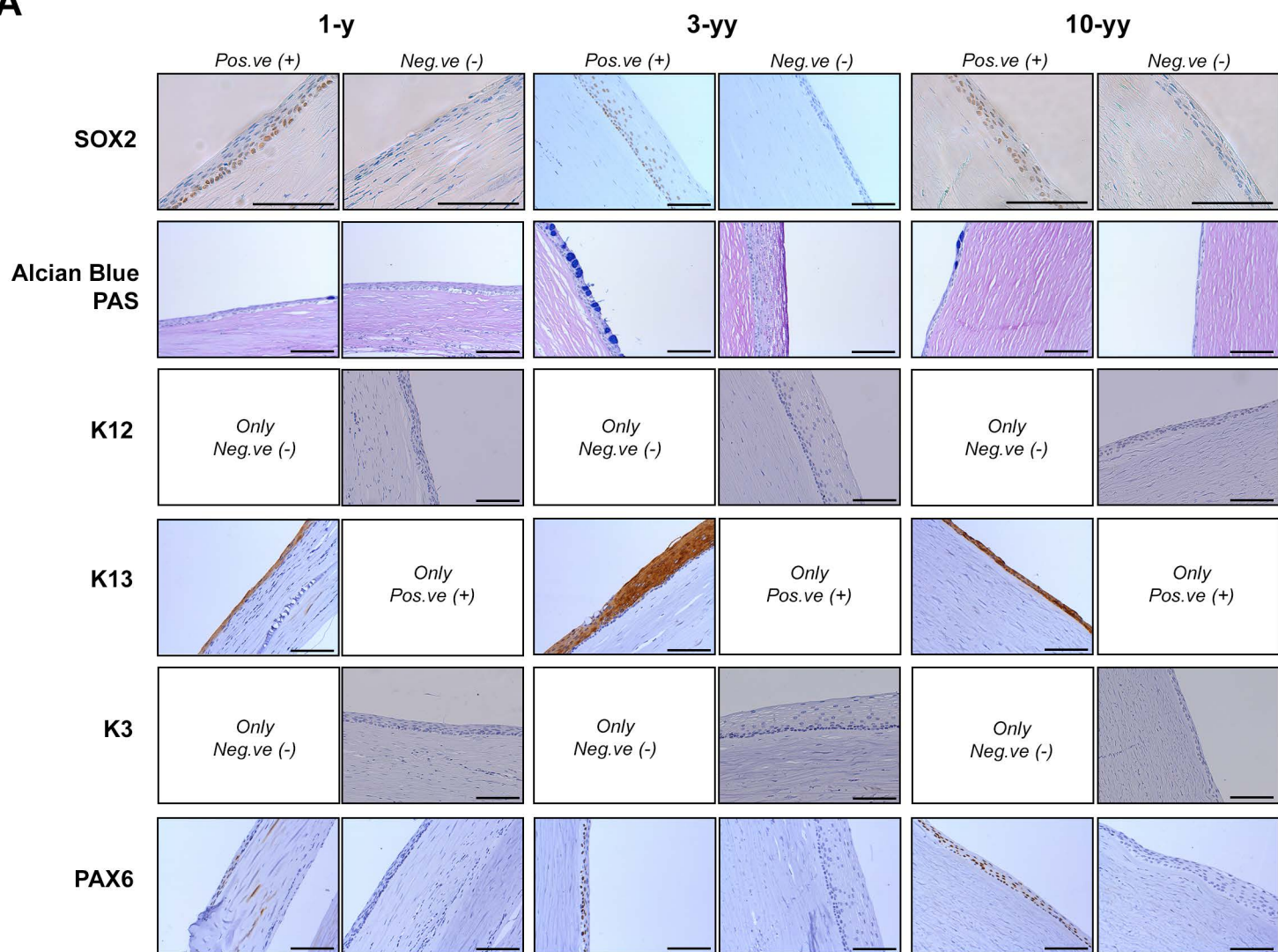**B**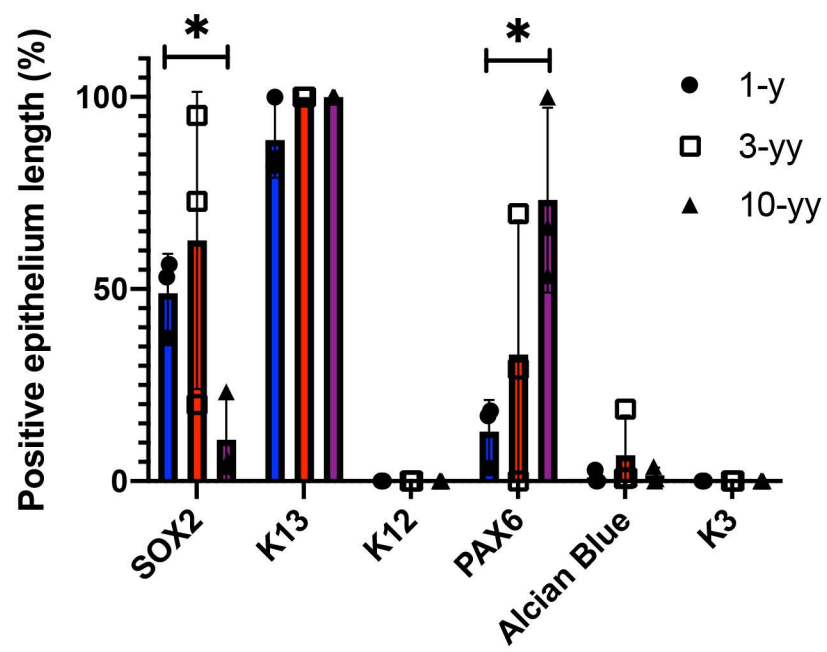

Supplement: Supplementary file 1 [file ijms-23-05785-s001.zip › Figure 4.pdf]

**A**

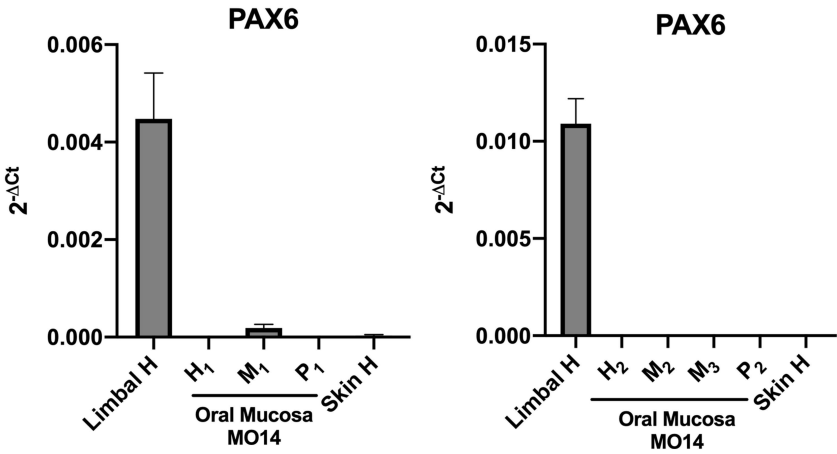

**B**

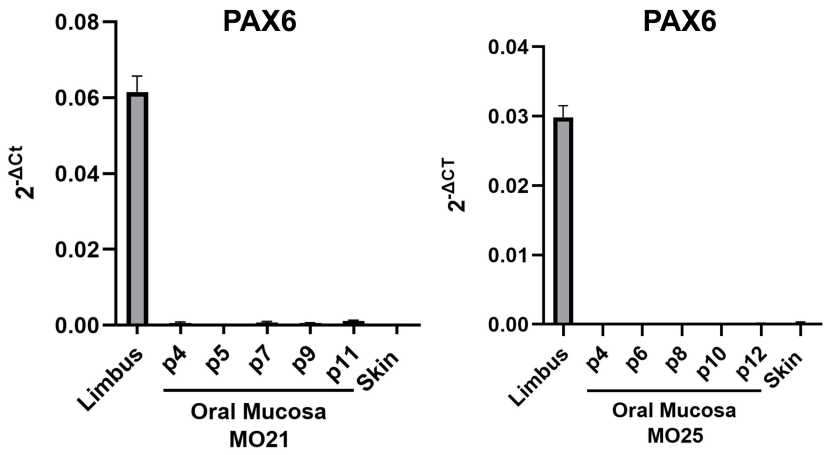

Supplement: Supplementary file 1 [file ijms-23-05785-s001.zip › Figure S1.pdf]
